# Health selection among outmigrants, return migrants and non-migrants in three subcohorts of international, interprovincial migrants and non-migrants in Manitoba, Canada

**DOI:** 10.23889/ijpds.v9i5.2792

**Published:** 2024-09-18

**Authors:** Amani Hamad, Marcelo Urquia, Gilles Detillieux, Shantanu Debbarman

**Affiliations:** 1 Manitoba Centre for Health Policy

## Objective and Approach

Linking national and provincial immigration registers with health care utilization datasets at the Manitoba Centre for Health Policy, we assembled a cohort of 816,185 adults who resided in Manitoba, Canada and were followed up for at least one year between 1985 to 2023 to outmigration, return migration or death. The cohort included three subcohorts of international immigrants (16.4%), interprovincial migrants (10.8%) and all other Manitobans (AOM) (72.8%). Within each subcohort, we matched ‘stayers’ who never migrated, outmigrants and returnees on sex, birth year and place of residence and compared their hospitalization rates and Charlson and Elixhauser comorbidity scores 1-year before outmigration and 1-year after return migration.

## Results

Outmigrants had lower hospitalization rates than stayers among AOM [Adjusted Relative Rate (RR): 0.80; 95% confidence interval (CI): 0.78, 0.82] and in the other two subcohorts. Comorbidity scores were also consistently lower among outmigrants compared to stayers in all three subcohorts, even after restricting to hospitalized cases. Outmigrants whose destination was another country were healthier than those who migrated to other provinces. Returnees had better health status than stayers upon return in the AOM subcohort only but lower than those who did not return at the time of outmigration in all three subcohorts.

## Conclusions

Migration is associated with positive health selection among international immigrants, interprovincial migrants and the local population.

## Implications

Contrary to common belief, health selection is not restricted to international immigrants. Selective migration may represent a source of bias in health-related population-based studies (e.g., sampling and informative censoring).

